# Microbial Diversity in Children with Gastroenteritis in the Amazon Region of Brazil: Development and Validation of a Molecular Method for Complete Sequencing of Viral Genomes

**DOI:** 10.7150/jgen.94116

**Published:** 2024-03-25

**Authors:** Juliana Merces Hernandez, Giovanna Brunetta SantAna Almeida, Ana Caroline Rodrigues Portela, Jedson Ferreira Cardoso, Edivaldo Costa Sousa Junior, Maria Silvia Sousa Lucena, Márcio Roberto Teixeira Nunes, Yvone Benchimol Gabbay, Luciana Damascena Silva

**Affiliations:** 1 Postgraduate Program in Biology of Infectious and Parasitic Agents, Federal University of Pará, Belém, Pará, Brazil.; 2 Scientific initiation fellowships program (PIBIC/CNPq), Evandro Chagas Institute, Ananindeua, Pará, Brazil.; 3 Center for Technological Innovation, Evandro Chagas Institute, Ananindeua, Pará, Brazil.; 4 Virology Section, Evandro Chagas Institute, Ananindeua, Pará, Brazil.

**Keywords:** parechovirus, metagenomics, complete genome

## Abstract

Introduction: Metagenomic sequencing is a powerful tool that is widely used in laboratories worldwide for taxonomic characterization of microorganisms in clinical and environmental samples. In this study, we utilized metagenomics to investigate comprehensively the microbial diversity in fecal samples of children over a four-year period. Our methods were carefully designed to ensure accurate and reliable results. Material and Methods: Validated and analyzed were metagenomic data obtained from sequencing 27 fecal samples from children under 10 years old with gastroenteritis over a four-year period (2012-2016). The fecal specimens were collected from patients who received care at public health facilities in the northern region of Brazil. Sequencing libraries were prepared from cDNA and sequenced on the Illumina HiSeq. Kraken-2 was utilized to classify bacterial taxonomy based on the 16S rRNA gene, using the Silva rRNA database. Additionally, the Diamond program was used for mapping to the non-redundant protein database (NR database). Phylogenomic analyses were conducted using Geneious R10 and MEGA X software, and Bayesian estimation of phylogeny was performed using the MrBayes program. The results indicate significant heterogeneity among norovirus strains, with evidence of recombination and point mutations. This study presents the first complete genome of parechovirus 8 in the region. Additionally, it describes the bacterial populations and bacteriophages present in feces, with a high abundance of Firmicutes and Proteobacteria, including an increased proportion of the *Enterobacteriaceae* family. The presented data demonstrate the genetic diversity of microbial populations and provide a comprehensive report on viral molecular characterization. These findings are relevant for genomic studies in gastrointestinal infections. The metagenomic approach is a powerful tool for investigating microbial diversity in children with gastroenteritis. However, further studies are imperative to conduct genomic analysis of identified bacterial strains and thoroughly analyze antimicrobial resistance genes.

## Introduction

Viral metagenomics is a powerful tool for discovering viruses. This methodology provides comprehensive genomic profiling to identify variants implicated in outbreaks of human and animal diseases. Next-Generation Systems' massive sequencing is a sequence-independent detection method that is highly effective in detecting viruses with high mutation rates.

It should be noted that viral gastroenteritis outbreaks are the second leading cause of death for children under five, resulting in 525,000 child deaths annually 5. Most infections in the human intestinal tract are caused by human viruses, resulting in approximately 200,000 deaths per year and accounting for over 90% of gastroenteritis outbreaks worldwide 6. Rotaviruses, noroviruses, and picornaviruses infect the human gastrointestinal tract and disrupt the complex microbial community composed of diverse biological components, including bacteria, archaea, eukaryotic microbes, and viruses 7,8.

The fecal microbial communities consist of four main phyla: Firmicutes, Bacteroidetes, Actinobacteria, and Proteobacteria. The virome is mainly composed of phages, specifically those of the Caudovirales order. The families *Myoviridae*, *Siphoviridae*, and *Podoviridae* are the most prevalent among viruses 9.

Monitoring the emergence and re-emergence of viral diseases is essential to identify genetic diversity and effectively pinpoint the causative agent of a new epidemic, enabling an effective response to disease outbreaks 10. The sequence-independent metagenomic approach has enabled the discovery of numerous viruses and bacteria in various samples. The approach highlights its power in uncovering novel and unique microorganisms, which are often distantly related to previously described microorganisms. The majority of the generated sequences do not resemble any known microorganisms 9, 10.

This study aims to investigate the microbial diversity in patients with gastroenteritis using metagenomics and validate a bioinformatics pipeline for taxonomic classification of bacteria and viruses present in fecal samples.

## Material and Methods

This study analyzed metagenomic data from fecal samples of children under 10 years old with gastroenteritis over a four-year period (2012-2016). The fecal specimens were collected from 27 inpatients who were attended to in public health facilities as part of the epidemiological surveillance studies of viral gastroenteritis by Instituto Evandro Chagas (Ministry of Health - Brazil). A sub-sample of gastroenteritis cases from the northern region of the country during the study period was selected for sequencing based on available information.

The study was approved by the Ethics Committee on Human Research of IEC (CAAE 46841715.8.0000.0019).

The fecal suspension (10% w/v) in Tris/HCl/Ca+2 buffer was extracted using the QIAamp mini kit (Qiagen, Hilden, Germany) following the manufacturer's guidelines. The Qubit RNA BR assay kit from Thermo Fisher Scientific and Qubit 2.0 fluorometer were utilized for nucleic acid quantification. Sample quality was evaluated using automated electrophoresis in the 2100 Bioanalyzer from Agilent Technologies, Santa Clara, CA, United States, following the manufacturer's recommendations.

The metagenomic analytics pipeline was designed and validated with confidence using reference samples. Testing was conducted only on reference samples with previously known results to ensure accuracy. *In silico* validation was performed using raw data from complete sequencing of positive norovirus samples available in GenBank.

Genomic libraries were prepared by performing cDNA synthesis using the cDNA synthesis system kit (Roche, Branford, CT, United States). To prepare sequencing libraries, we used the Illumina Nextera XT DNA Library Prep Kit to process 5 ng of DNA (or cDNA). The libraries were then sequenced on the Illumina HiSeq 2500 instrument using the high-output V4 2x100-bp sequencing kit (Illumina, San Diego, CA, United States).

After sequencing, the raw reads underwent quality control and adapter removal using the Trim_Galore tool. Additionally, low-quality sequencing tails were trimmed using a Phred quality score of 30.

Bacterial taxonomic classification was performed using Kraken-2, which utilizes k-mer matches for rapid classification. The classification was based on the 16S rRNA gene with the Silva rRNA database 12. Taxonomic classification was confidently performed using the Diamond program and mapped to the NCBI non-redundant protein database. The genome assembly was carried out using the IDBA-UD algorithm, which is based on the de Bruijn graph and supports unequal coverage depth, resulting in longer and more accurate contigs 13. The Krona tool was used to generate graphical plots, allowing for interactive data visualization and accurate estimation of microorganism abundance 14.

Comparative genomics and phylogenomic analysis were conducted using the Geneious R10 program 15. The final database consisted of sequences with 80-100% identity (Blastn). The norovirus and enterovirus genotypes were confirmed with high confidence using the Norovirus Genotyping Tool, BLAST (Basic Local Alignment Search Tool), and Enterovirus Genotyping Tool (https://www.rivm.nl/mpf/typingtool).

The Gegeenes program was utilized to perform comparative genomic analyses and investigate specific differences between genomes. The program determined the phylogenomic distances and genetic signatures present in the samples through a fragmented alignment of the available norovirus genomes 16.

To provide a comprehensive analysis of genomic diversity, phylogenomic analyses were conducted with confidence and precision. The sequences were aligned using the Muscle alignment tool. Phylogenetic reconstructions were performed using maximum likelihood methods and the GTR + I + G4 nucleotide substitution model calculated by jModelTest v. 2.1 with 1000 bootstraps. Evolutionary distances were calculated using the Kimura 2-parameter model. Additionally, an evolutionary analysis was conducted using the MrBayes program for Bayesian estimation of phylogeny.

The biostatistics analyses were performed using Bioestat 5.0 software. The statistical analyses conducted included the Shannon-Wiener Index, Spearman correlation coefficient, and simple logistic regression analysis. A p-value less than 0.05 was considered statistically significant.

## Results

This study used whole genome sequencing to assess microbial diversity in fecal samples from children under 10 years old diagnosed with gastroenteritis.

The sequencing generated around 317 million paired-end reads, with each sample ranging from 6.3 to 16.5 million reads. A total of 60,564,491 contigs were assembled using binning methods. The majority of these contigs belonged to the Bacteria domain (57,540,287 counts, 95%), followed by Eukaryota (1,885,240 counts, 3.1%) and Viruses (1,138,964 counts, 1.9%).

The analysis strategy that proved to be the most accurate for viral contigs involved mapping with the NR database, assembly with IDBA-UD, and taxonomic classification using Diamond. This study identified 14 viral genomes from the *Caliciviridae* and *Picornaviridae* families. Kraken 2 was found to be a more accurate and efficient tool for identifying bacterial genomes, requiring fewer computational resources than IDBA-UD.

**Figure 1:**
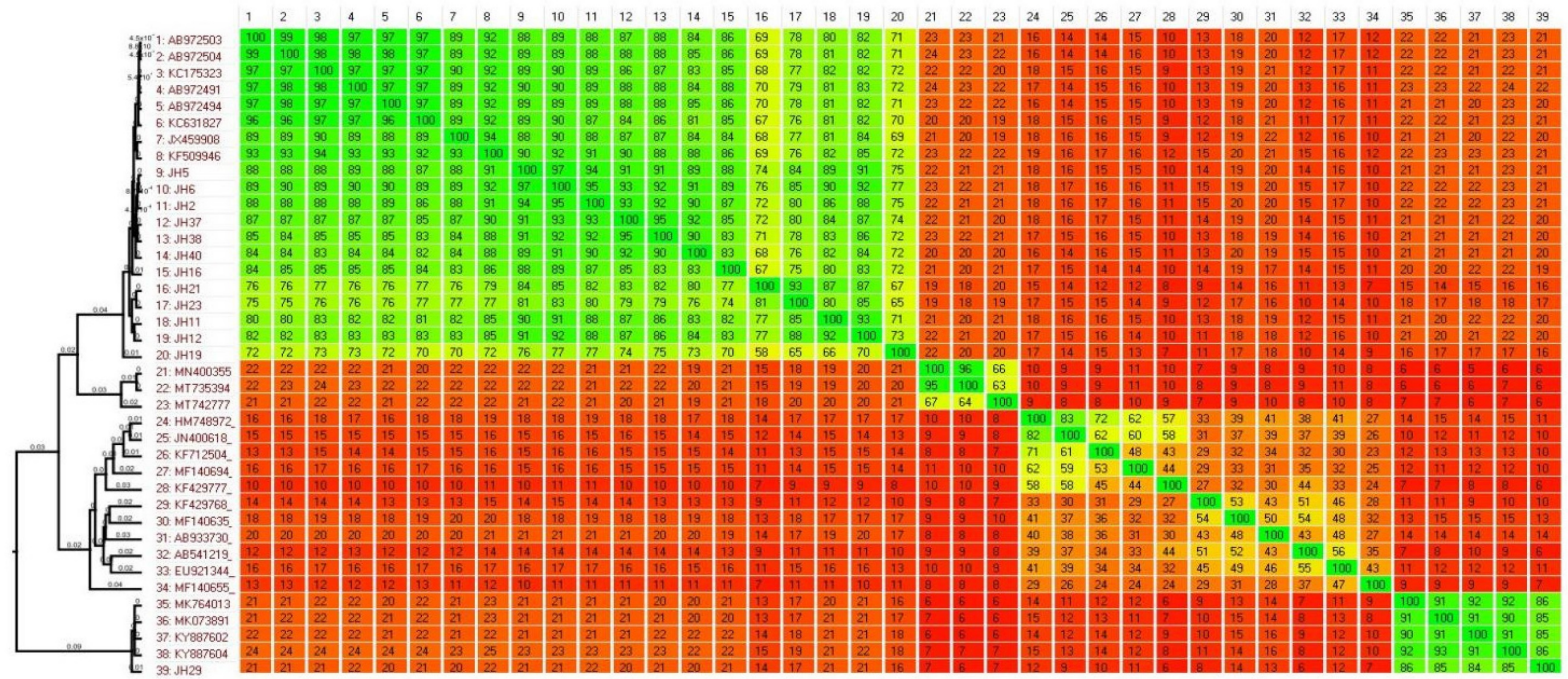
Comparative analysis of whole genome sequence data performed using the Gegeenes software. The heat-plot was based on a fragmented alignment using BLASTN with settings 50/25. A dendrogram was produced in SplitsTree 4 using the neighbor joining method from a Nexus file exported from Gegenees.

**Figure 2:**
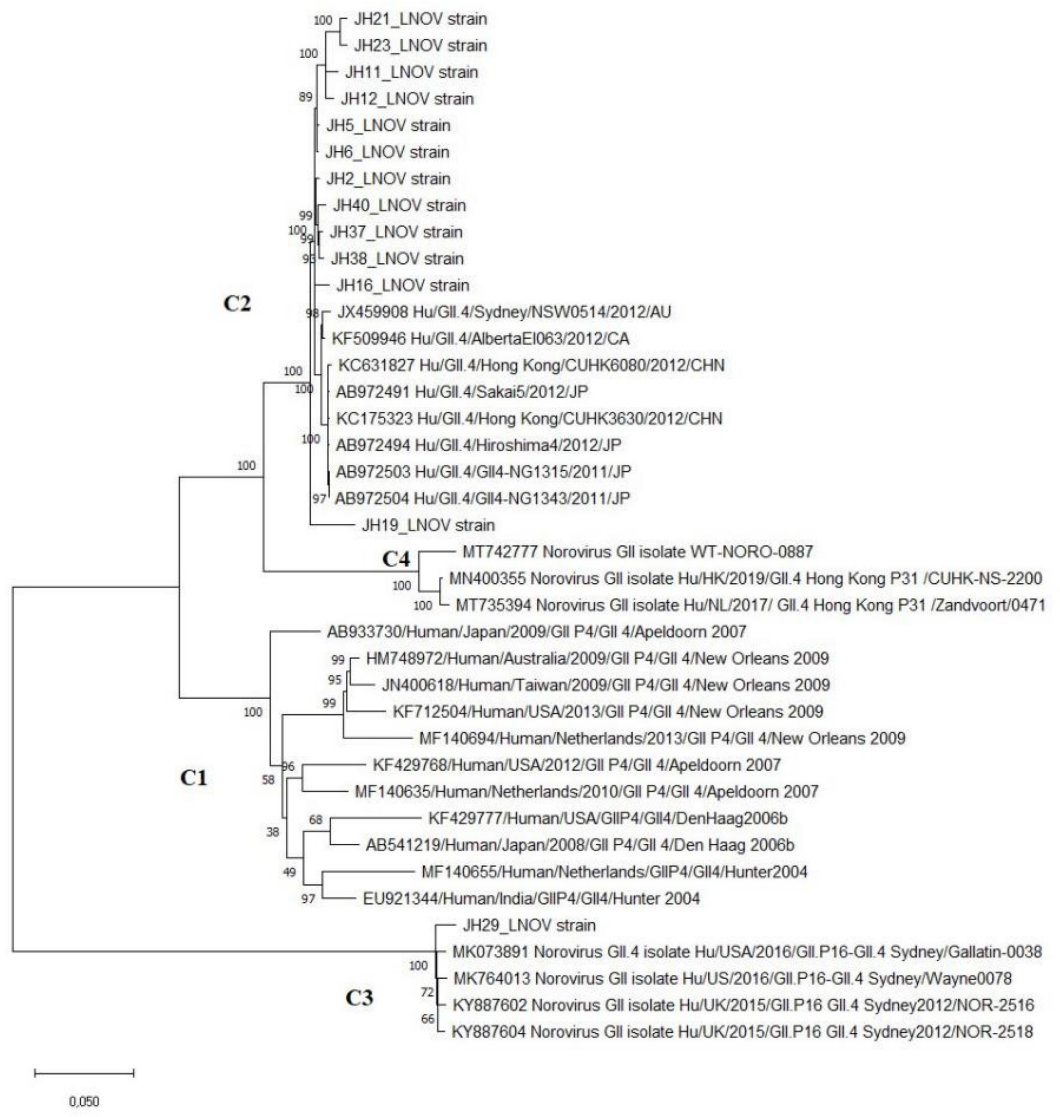
Phylogenomic tree of norovirus reconstructed by evolutionary analysis by Maximum Likelihood method. The tree is drawn to scale, with branch lengths measured in the number of substitutions per site. This analysis involved 39 nucleotide sequences in MEGA X.

The phylogenomic analysis of norovirus strains revealed heterogeneity when compared to previously circulating strains. The phylogenies and heatplot demonstrate that GII4 strains can be categorized into distinct clusters based on their circulation period and pairwise distances (Figure 1). The homology ranges for intercluster and intracluster were 70-92% and 65-97%, respectively. The ML tree identifies four distinct clusters of GII4 variants (C1-C4). The C1 cluster comprises old GII4 variants that circulated from 2004 to 2010, such as Hunter_2004, Den-Haag_2006, Alperdoon_2007, and New Orleans_2009. The C2 cluster consists of the GIIP31/GII4 variant, while the C3 cluster represents the GIIP16/GII4 that emerged in 2015/2016. Finally, the C4 cluster includes the GII4 Hong Kong strain, which was detected in a sample from 2019 (Figure 2).

Recombination analyses confirm that the GIIP.16/GII.4 strain originated from a recombination event between the GIIP31/GII4 and GIIP16 genotypes. The bootscan analysis clearly identified a recombination breakpoint between ORF1 and 2. Although these strains share similarities in the ORF2 region, the differences, mainly present in ORF1, are crucial for phylogenetic segregation.

**Figure 3:**
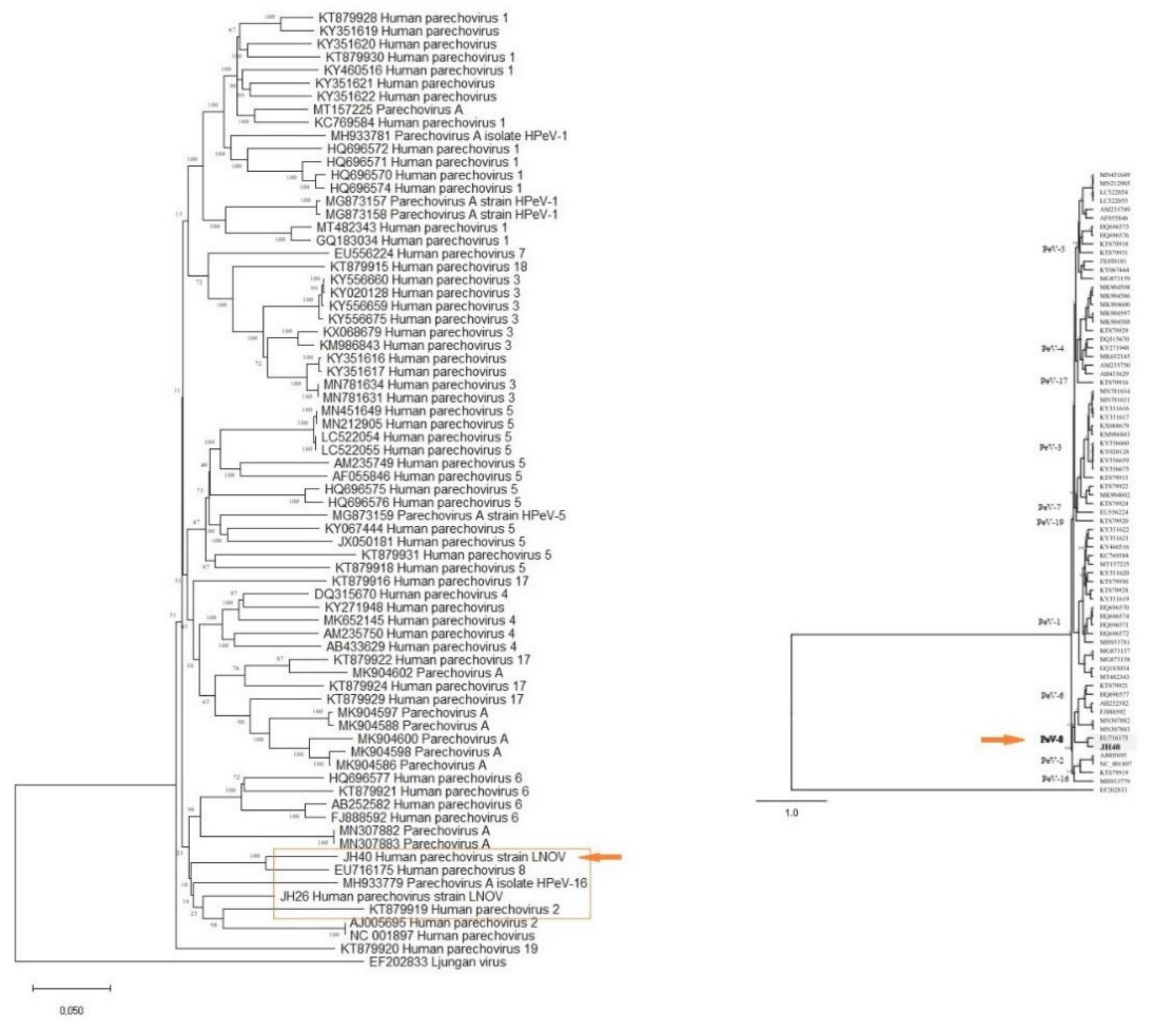
** (A)** Phylogenomic tree of parechovirus. The evolutionary distances were computed using the Maximum Composite Likelihood method and are in the units of the number of base substitutions per site. This analysis involved 83 nucleotide sequences in MEGA X. **(B)** A phylogenetic tree of the complete genome of parechovirus created using Bayesian inference in the Geneious software with MrBayes. The program produced consensus trees and partition tables summarizing the trees and branch lengths. The consensus trees were written with both branch lengths and posterior clade probabilities.

**Figure 4:**
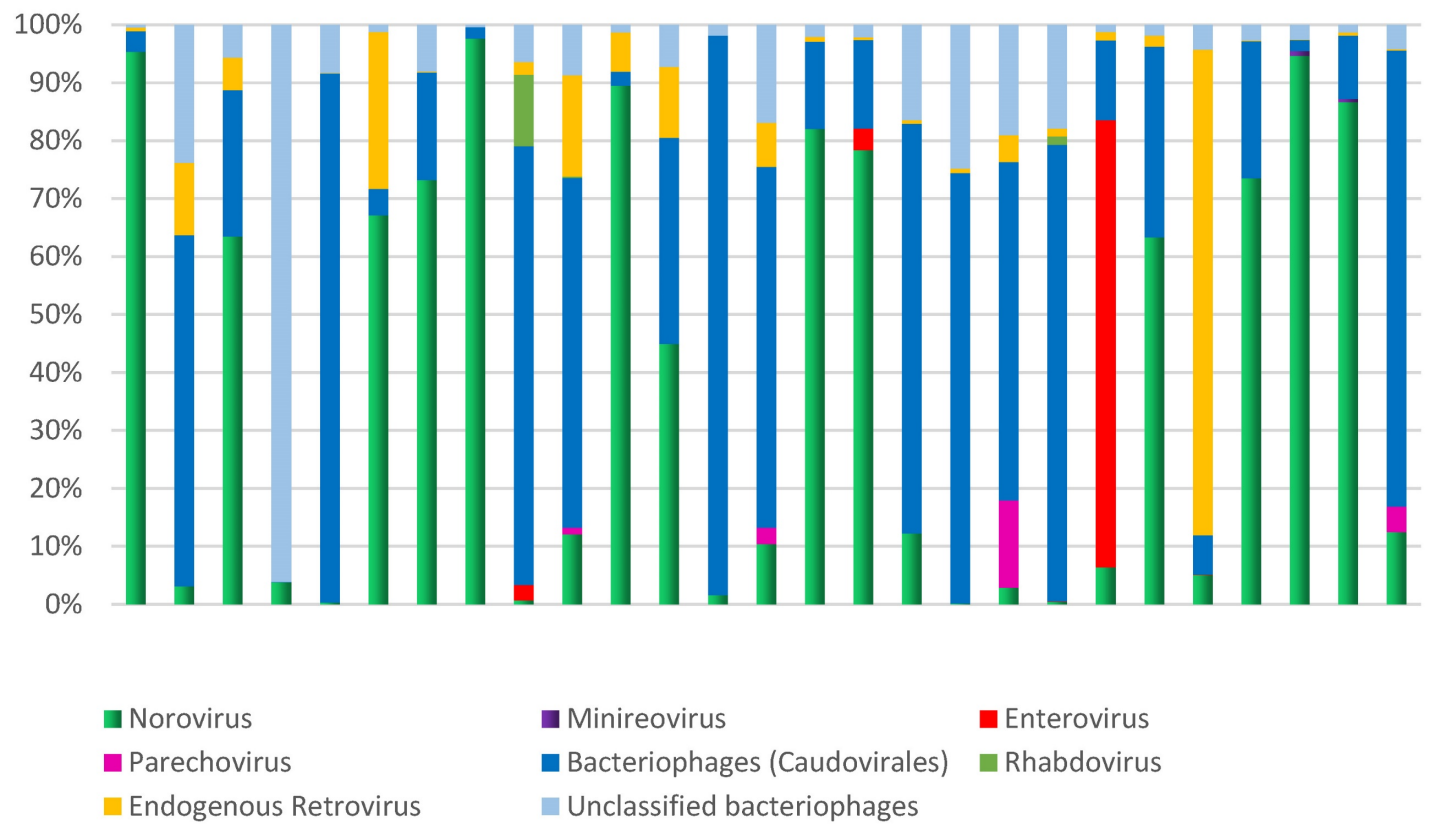
Microbial diversity in children hospitalized with gastroenteritis in the northern region of Brazil. The analysis focused on the relative demonstration of the components of the virome in each sample.

Additionally, two strains of parechovirus A, specifically HPeV-2 and HPeV-8, were detected (Figure 3). One of these strains (JH40) has had its complete genome sequenced, revealing a length of 7190 pb and 87.27% homology to strain EU716175, which was first identified in Brazil in 2009. Notably, this strain (PeV/LNOV_2013) has an evolutionary change rate of 5.7 x 10^-5^ substitutions/site/year (Figure 4). It is worth noting that no other representatives of the Parechovirus genogroup 8 were found in Genbank. A matrix of patristic distances was calculated from a NJ tree to summarize the phylogenetic changes between the two Parechovirus 8 strains.

Phylogenetic differences between PeV/LNOV_2013 and EU716175 were minimal, with distances of 0.05, 0.06, and 0.07 for VP3, VP1, and VP0, respectively. Patristic distances in regions 2ABC and 3ABC ranged from 0.4-0.5. There is no evidence of recombination with other prototype strains in these nonstructural gene regions.

**Figure 5:**
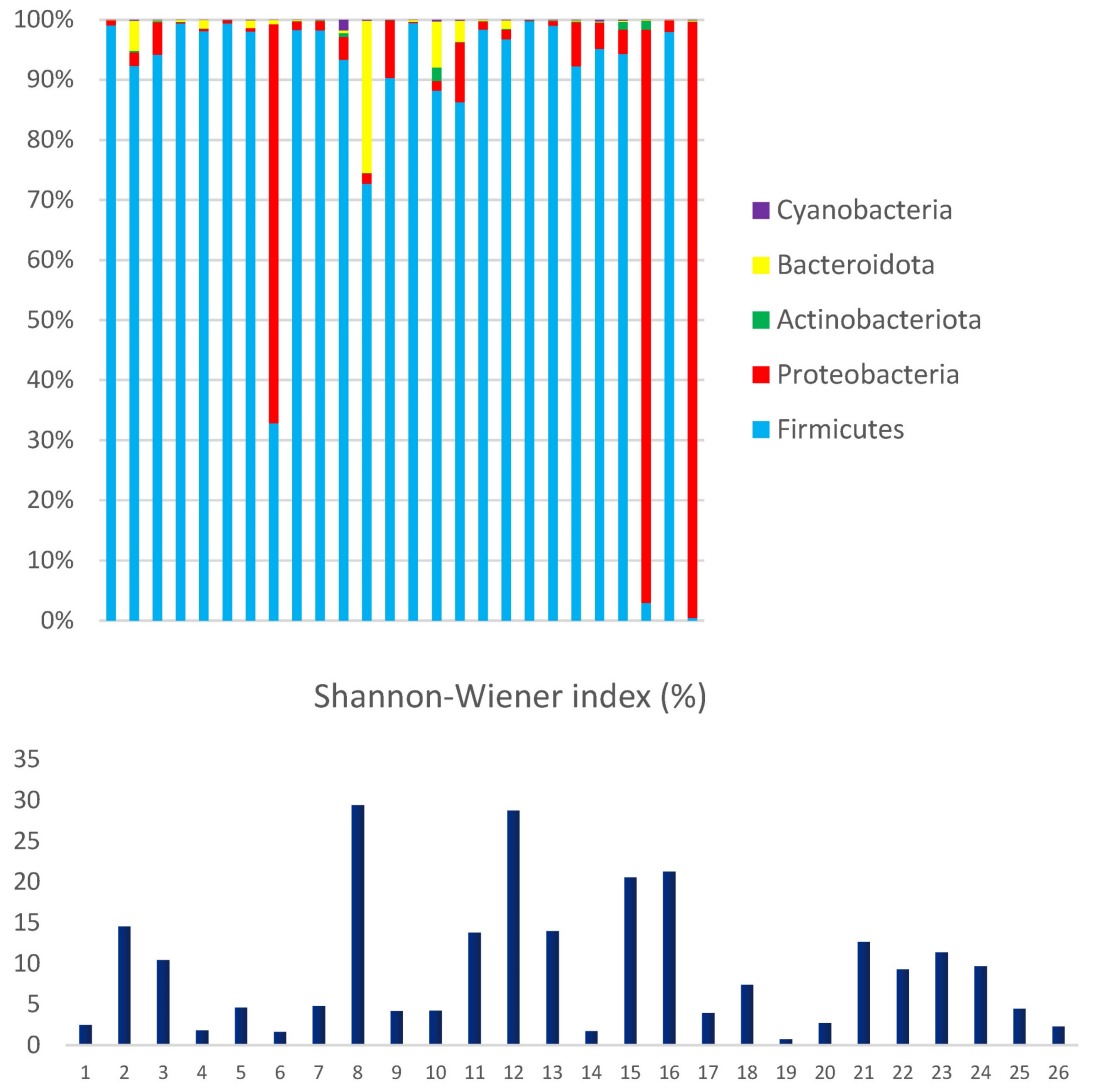
Microbial diversity in children hospitalized with gastroenteritis in the northern region of Brazil. Relative demonstration of the bacterial phyla that comprise the human fecal microbiome and compares it to Shannon-Wiener's index.

Additionally, other components of the virome, such as minireovirus, rhabdovirus, and unclassified bacteriophages, were also found (Figure 5). The analysis of the samples revealed frequent observations of bacteriophages and human endogenous retroviruses. Abundant bacteriophages of the order Caudovirales were found, specifically from the families *Siphoviridae*, *Podoviridae*, and *Myoviridae*.

The most abundant phyla among the Bacteria domain were Firmicutes (86.3%) and Proteobacteria (12.5%). Actinobacteriota, Bacteroidota, and Cyanobacteria were less represented, accounting for only 1.2% of the mapped reads. The interindividual variations in microbial composition were estimated using Shannon Wiener's index, which evaluates richness and equitability.

The observed high interindividual variation reflects microbial diversity and can be driven by the abundance of dominant phyla (Figure 5). The data clearly demonstrate a significant disequilibrium, primarily due to the high proportion of Proteobacteria (above 5%) observed in 27% (7/26) of the analyzed samples. A statistical analysis was conducted on bacterial phyla, revealing a strong correlation between the proportions of Proteobacteria and Actinobacteriota (R-Pearson= 0.5614, p=0.0028). Additionally, a significant correlation was found between the phyla Bacteroidota and Cyanobacteria (R-Pearson= 0.5191, p=0.0066).

The taxonomic profile of the analyzed samples was thoroughly examined through a relative study, which identified the main bacterial families present in human fecal metagenomes. The study compared the results to data from other studies, which further solidified the findings. The study identified several bacterial families, including *Enterobacteriaceae*, *Bacteroidaceae*, *Rikenellaceae*, *Tannerellaceae*, *Prevotellaceae*, *Flavobacteriaceae*, and *Porphyromonadaceae*, all belonging to the Proteobacteria and Bacteroidetes phyla.

The *Enterobacteriaceae* family was the predominant group, comprising 89.5% of the total, with Klebsiella being the most prevalent genus at 91.4%. The sample contained 3.7% of other enterobacteria, while Escherichia-Shigella, Salmonella, and Enterobacter accounted for 1.8%, 0.4%, and 2.7%, respectively. *Bacteroidaceae*, *Prevotellaceae*, and *Flavobacteriaceae* were also detected, representing 2%, 3.3%, and 5% of mapped reads, respectively. *Rikenellaceae*, *Tannerellaceae*, and *Porphyromonadaceae* were present at a combined proportion of 0.2% of reads. The analysis confirms the presence of potential pathogenic bacteria, with Klebsiella being the most abundant.

Additionally, the viral sequences have been successfully deposited in the GenBank database (accession numbers: MT 474032.1 - MT 474035.1, MT 474037.1 - MT 474038.1, and MT 474040.1 - MT 474052.1).

## Discussion

The metagenomics approach allows for molecular surveillance of human infection-causing pathogens, aiding in the recovery of genomes for outbreak identification and molecular characterization 21.

The viral strains identified in this study were GIIP31/GII.4 and GIP16.GII4 norovirus genotypes, as well as HPeV-2 and HPeV-8 parechovirus genotypes. Phylogenetic trees were reconstructed based on the whole genome data instead of a single gene. Two approaches were used to confidently reconstruct phylogenomic trees and infer the evolutionary history between norovirus GII4 strains and parechovirus 8 strains: sequence-based and gene content-based.

The GIIP31/GII.4 and GIP16.GII4 strains have been continuously evolving in the region since 2013 and 2016, respectively, and have been responsible for the majority of norovirus cases detected 22. The primary driving forces behind genetic diversity in viruses are recombination and point mutations. These processes lead to the evolution of viral strains. Recombination occurs in the overlapping region of ORF1 and ORF2, resulting in strains with distinct genotypes in the polymerase and capsid regions 23.

HPeV infections primarily affect children and are associated with respiratory and gastrointestinal symptoms. These viruses are increasingly recognized to be excreted long-term in stool. The epidemiology and clinical manifestations of Human Parechovirus (HPeV) infections vary depending on the virus genotype. HPeV1 and HPeV6 are commonly associated with gastroenteritis, while HPeV3 and HPeV5 are typically associated with sepsis-like illness 24, 25.

Our study found HPeV8 in a child with gastroenteritis, which is consistent with other studies conducted in Brazil. The molecular epidemiology of these viruses is a significant factor in their diverse and strain-dependent pathogenesis 25. In Brazil, the genotypic distribution of human Parechovirus (HPeV) is heterogeneous, with not all genotypes being detected in the same locality. This report confirms that HPeV8 is a cause of gastroenteritis in Brazilian children and is distributed throughout the country.

The VP1 region is utilized to distinguish between various Parechovirus genotypes based on phylogenetic differences. This region undergoes a high rate of evolutionary change, with approximately 10^-3^ substitutions per site per year, according to Bayesian analysis. It is highly probable that Parechovirus A species diverged from their most recent common ancestor around 400 years ago and have since evolved into different lineages. The study reports that the PeV/LNOV_2013 strain detected in this research evolves at a rate of 5.7 10^-5^ substitutions/site/year and diverges by 12.73% from HPeV-8, which was first described in Salvador in 2006 25. The fecal samples of the children enrolled in this study contained high levels of Firmicutes and Proteobacteria, as well as several pathogenic bacteria, including Escherichia, Shigella, Salmonella, Klebsiella, and Enterobacter. The *Enterobacteriaceae* family was the most abundant. These findings are in line with prior research indicating high levels of enterobacteria 26, 27. Chen et al. (2017) found a significant correlation between the presence of the *Enterobacteriaceae* family and gastroenteritis in patients compared to a control group 28.

The even distribution of microorganisms in the analyzed samples, as demonstrated by the Shannon-Wiener diversity index, suggests that these individuals had similar intestinal flora. Previous studies have shown that gastroenteritis reduces microbial diversity, as demonstrated by the entropy score 28.

The main bacterial phyla in the intestine, identified by previous research on microbial diversity, are Firmicutes and Bacteroidetes, which together comprise over 90% of the total community. Actinobacteria, Proteobacteria, and Verrucomicrobia follow in abundance 29, 30. The gut microbiota's taxonomic profile indicates that the Firmicutes bacterial phylum is the most abundant, followed by Proteobacteria, Actinobacteriota, Bacteroidota, and Cyanobacteria. This suggests a disruption of the gut microbiota, which can be caused by pathogenic bacteria and viruses, resulting in an enhanced Firmicutes-to-Bacteroidetes (F/B) ratio in the gut 29.

The study used a metagenomic approach to assess the microbial diversity in children with gastroenteritis. The results clearly showed the prevalence of enterobacteria in conjunction with enteric viruses, such as noroviruses and parechoviruses. Additionally, the study noted the coexistence of bacterial populations and their viruses (bacteriophages). These findings suggest that metagenomics is a valuable tool for identifying microbial diversity in fecal samples.

This investigation provides a report on the genomic characterization of viral strains and the microbial diversity in children from the Amazon region. Future studies should include genomic analysis of the identified bacterial strains and an analysis of antimicrobial resistance genes.
